# Species Composition and Biomass Dynamics of Filamentous Algae and Their Environmental Drivers in *Eriocheir sinensis* Aquaculture Ponds

**DOI:** 10.3390/biology15030286

**Published:** 2026-02-05

**Authors:** Yudi Song, Fei Fei, Dijun Luo, Jie Yang, Gaohua Ji, Xugan Wu

**Affiliations:** 1 National Demonstration Center for Experimental Fisheries Science Education, Shanghai Ocean University, Shanghai 201306, China; 2 Chongming District Aquatic Technology Extension Station, Shanghai 202150, China; 3 Research Centre of the Ministry of Agriculture and Rural Affairs on Environmental Ecology and Fish Nutrition, Shanghai Ocean University, Shanghai 201306, China

**Keywords:** sustainable aquaculture management, pond ecosystem, environmental drivers, crab aquaculture

## Abstract

Filamentous opportunistic algae, which behave as opportunistic species, are frequently observed in *Eriocheir sinensis* aquaculture ponds. These algae can entangle *Eriocheir sinensis* and release harmful substances during decomposition, thereby negatively impacting farming performance. At present, their management largely depends on non-selective herbicides, while fundamental research on species composition and biomass dynamics remains limited. In this study, 19 aquaculture ponds across five *E. sinensis* farms in Shanghai were monitored over a two-year period. Filamentous algae species were identified using both morphological and molecular techniques, and their biomass and coverage were quantified. Concurrently, physicochemical parameters of the water were measured to analyze algal occurrence patterns and key environmental drivers. A total of 19 species belonging to four genera of the phyla *Chlorophyta* and *Charophyta* were identified. *Rhizoclonium* was the most common genus, followed by *Cladophora* and *Spirogyra*. These genera exhibited distinct seasonal succession, with *Cladophora* and *Spirogyra* dominating in spring, while *Rhizoclonium* predominanted in summer and autumn. Filamentous algal biomass reached its peak in May 2024, with a dry weight of 42.92 g/m^2^. The two-way ANOVA results indicated significant main effects of month and region, as well as a significant month × region interaction. The Spearman correlation analysis revealed a strong positive association between algal biomass and pH. This pattern is consistent with the effect where the intense algal photosynthesis raises water pH through the uptake of dissolved carbon dioxide. The total biomass was significantly correlated with the nitrogen-to-phosphorus ratio, suggesting that nitrogen and phosphorus availability influenced algal growth. Moreover, filamentous algal coverage was positively associated with maximum algal biomass. The linear regression analysis further revealed that multiple environmental factors jointly contributed to algal proliferation, with total nitrogen, nitrate nitrogen, and fluorescent dissolved organic matter (fDOM) showing relatively strong associations with maximum biomass. These findings provide a scientific basis for the ecological control and targeted management of filamentous algae in aquaculture systems.

## Simple Summary

This study investigates the blooms of filamentous algae that are frequently observed in *Eriocheir sinensis* aquaculture ponds. The excessive growth can form dense green mats along pond margins and at the water surface, hindering crab movement, degrading water quality, and increasing management difficulty. We monitored 19 ponds across five farms for 2 years. The filamentous algae were identified using microscopy and genetic analyses, and we tracked seasonal changes in algal coverage and biomass together with environmental factors. Nineteen species of filamentous algae belonging to four genera were recorded. Filamentous algal biomass and coverage varied markedly among ponds and years and were associated with multiple environmental factors. These findings provide a scientific basis for integrated pond management and for controlling filamentous algal blooms in the *Eriocheir sinensis* aquaculture systems.

## 1. Introduction

*Eriocheir sinensis*, commonly known as the Chinese mitten crab, is one of the most important freshwater aquaculture species in China, with an annual production of approximately 800,000 tons [1]. In recent years, filamentous algal blooms have become a major problem in aquaculture systems, particularly in ponds used for *Eriocheir sinensis* and crayfish farming.

Filamentous algae in aquaculture ponds are mainly composed of filamentous taxa from the phyla Chlorophyta and Charophyta, along with filamentous forms from Cyanobacteria, including typical genera such as *Cladophora*, *Spirogyra*, and *Rhizoclonium*. On the one hand, these algae can play a beneficial role in aquaculture systems. During their growth, they assimilate nutrients and perform photosynthesis, thereby increasing dissolved oxygen levels and improving water quality. Some attached large filamentous algae are also capable of absorbing and degrading inorganic and organic pollutants, contributing to the maintenance of ecosystem structure and function [2].

On the other hand, the excessive proliferation of filamentous algae can have substantial negative impacts on the *Eriocheir sinensis* aquaculture. Dense mats covering the pond surface limit light penetration into deeper water layers, inhibit photosynthesis of submerged macrophytes and phytoplankton, and lead to the simplification of algal communities, thereby impairing key ecological functions such as primary production, nutrient cycling and habitat provision within the pond ecosystem [3,4]; they also reduce habitat complexity and directly threaten crab growth and survival. Moreover, filamentous algae compete with phytoplankton for nitrogen and phosphorus, disrupting nutrient balance and potentially promoting harmful cyanobacterial blooms [5,6]. These cascading effects degrade the rearing environment, threaten crab health and survival, and initiate a reinforcing cycle of intensified algal competition and declining water quality. The decay of accumulated filamentous algal biomass increases oxygen demand and can promote hypoxic or anoxic conditions; under such environments, microbial processes may generate and release reduced compounds, including hydrogen sulfide (H_2_S) and ammonium, thus worsening water quality [7]. In addition, physical entanglement with filamentous algae can hinder crab respiration, locomotion, and molting [8,9].

The occurrence and proliferation of filamentous algae are regulated by multiple environmental factors, including water temperature, light, nutrient load, and microbial interactions [10,11]. Temperature strongly influences the colonization process and seasonal succession [12,13], while light intensity affects pigment formation, spore germination, and growth [14,15]. Nutrient levels, particularly nitrogen and phosphorus levels, also play a central role. The nitrogen-to-phosphorus ratio is widely used to infer potential nutrient limitation. N-deficient growth was generally observed when TN:TP was below 20, whereas P-deficient growth was consistently observed when TN:TP exceeded 50; at intermediate ratios, either N or P can become deficient [16].

Previous studies have primarily focused on filamentous algae in natural freshwater systems, such as *Cladophora glomerata* in European lakes [17] and eutrophic lakes in North America [18,19]. These investigations have revealed the combined effects of nutrient imbalance and trace elements (such as Fe^2+^) on the proliferation of filamentous algae, as well as the molecular mechanisms underlying microbe–algae interactions, including the algicidal activity of specific bacterial taxa [20]. In terms of control strategies, Li (2013) compared the effects of Cu^2+^, Zn^2+^, and Pb^2+^ on the growth of *Cladophora* and found that all three heavy metals inhibited growth, with the inhibitory strength in the order Zn^2+^ > Cu^2+^ > Pb^2+^ [21]. In aquaculture practice, the overgrowth of filamentous algae is often managed through the application of chemical algicides, which can cause irreversible ecological damage and secondary pollution [22].

Despite extensive studies in natural freshwater systems, filamentous algae dynamics in *E. sinensis* aquaculture ponds—a highly managed, semi-enclosed freshwater ecosystem—remain poorly understood. Specifically, it is unclear which species dominate, how their biomass and spatial distribution change over time, and which environmental factors most strongly drive their proliferation. Addressing these questions is critical for developing ecologically sound management strategies in aquaculture.

To provide insights into these knowledge gaps, this study conducted a two-year field investigation of *E. sinensis* aquaculture farms in Chongming, China, to (1) analyze the species composition, spatiotemporal distribution, and biomass dynamics of filamentous algae and (2) identify key environmental factors influencing their proliferation. The findings provide a scientific basis for the ecological management and sustainable restoration of *E. sinensis* aquaculture ponds.

## 2. Materials and Methods

### 2.1. Study Area and Sampling Site Setup

**Figure 1:**
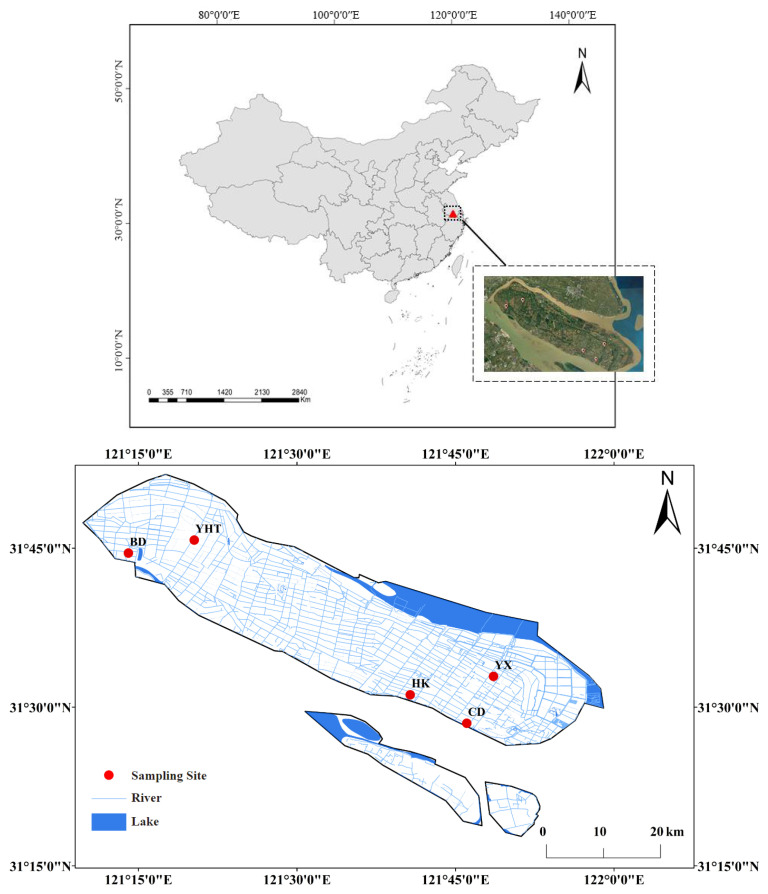
The distribution of sampled aquaculture farms in Chongming, China.

Five aquaculture farms, namely Chongdong Aquaculture Farm (CD), Huikang Aquaculture Farm (HK), Yuhaitang Aquaculture Farm (YHT), Baodao Farm (BD), and Yuxi Aquaculture Farm (YX) were selected, comprising a total of 19 ponds (Figure 1). These ponds were surveyed monthly from March (spring) to October (autumn) in 2023 and 2024. The *Eriocheir sinensis* aquaculture cycle lasts about 7 months, with juvenile crabs stocked in March and harvested in October of the same year.

The detailed characteristics of all aquaculture ponds are summarized in Table 1.

### 2.2. Sample Collection and Processing

#### 2.2.1. Qualitative Sampling of Filamentous Algae

Four to five random samples of filamentous algae within a 1 m^2^ area were collected from around the pond. Samples were kept in an ice box and transported to the laboratory, stored at 4 °C, and identified within 24 h.

Morphological identification was performed under an optical microscope, and photos were taken to record the normal cell and spore morphology, as well as filament structure. For molecular identification, representative filaments were isolated under a dissecting microscope and preserved in 95% ethanol for DNA extraction (Shanghai Maipu Biotechnology Co., Ltd., Shanghai, China). The remaining samples were stored in 4% formalin for long-term preservation. The internal transcribed spacer (ITS) region was amplified using the primers Clado ITS-9F and Clado ITS-7R, following the protocol described by Hayakawa [23]. Genomic DNA was extracted using standard protocols (Shanghai Maipu Biotechnology Co., Ltd.). DNA concentration and purity were assessed using a UV spectrophotometer, ensuring concentrations within the range of 20~30 ng/µL. The extracted DNA was stored at −20 °C. PCR amplification of the ITS region was performed with CladoITS-9F (5′-CCGCCCGTCGCTCCTACCGATTGGGTGTG-3′; 29 nt) and CladoITS-7R (5′-TCCCTTTTCGCTCGCCGTTACTA-3′; 23 nt) primers (PAGE-purified) under the following thermo-cycling program: 94 °C for 5 min; 35 cycles of 94 °C for 30 s, 50 °C for 30 s, and 72 °C for 1 min; followed by 72 °C for 10 min. The PCR products were purified and sequenced on an ABI 3730xl DNA Analyzer (Applied Biosystems, Foster City, CA, USA).

#### 2.2.2. Hydrochemical Analysis

Water samples were collected simultaneously for physicochemical analyses. Total nitrogen (TN) was determined by alkaline potassium persulfate digestion followed by ultraviolet spectrophotometry. Total phosphorus (TP) was measured using the ammonium molybdate colorimetric method. Dissolved total phosphorus (DTP) was determined in filtered samples through potassium persulfate digestion, followed by ammonium molybdate colorimetry. Ammonia nitrogen (NH_3_-N) was determined through the Nessler reagent spectrophotometry. Nitrate nitrogen (NO_3_^−^-N) was determined through ultraviolet spectrophotometry, and nitrite nitrogen (NO_2_^−^-N) was determined through N-(1-naphthyl)-ethylenediamine spectrophotometry. Suspended solids (SS) were determined gravimetrically. Soluble reactive phosphorus (SRP) was determined through the ammonium molybdate colorimetric method. The permanganate index (COD_Mn_) was measured using the acidic potassium permanganate method. In situ hydrochemical variables were recorded at each pond using the YSI EXO2 multiparameter water quality meter (YSI Inc., Yellow Springs, OH, USA), including water temperature (WT), pH, dissolved oxygen (DO), chlorophyll a (Chl-a), turbidity (NTU), conductivity (Cond), specific conductance (SpCond), total dissolved solids (TDS), salinity (Sal), fluorescent dissolved organic matter (fDOM), and cyanobacterial phycocyanin fluorescence (BGA-PC).

#### 2.2.3. Quantitative Sampling of Filamentous Algae

The frequency (%) was calculated in the following way: each taxon was divided by the total number of sequenced samples in this study (*n* = 39) and multiplied by 100.

The occurrence rate (%) was calculated from monthly genus records for each sampling station. For each station and sampling month, each recorded genus contributed one monthly record; when two genera were recorded in the same month, each genus was counted once. The occurrence rate for a given genus was calculated as the number of its monthly records divided by the total number of monthly records for that station, expressed as a percentage.

A drone (DJI Mavic 2 Pro, Dajiang Innovation Technology Co., Shenzhen, China) was used to capture aerial photographs (110~140 m altitude) of each pond. The images were processed with Image J software (v1.53t; National Institutes of Health, Bethesda, MD, USA) to delineate and calculate the area covered by filamentous algae. The coverage was determined using the following formula:

Coverage (%) = Area of filamentous algae/Total area × 100%(1)

Three random 0.5 m × 0.5 m quadrats were selected in each pond. Filamentous algae were collected from each quadrat, impurities were removed, and the samples were placed in sealed bags under low temperature for transport. The samples were cleaned, surface water removed using absorbent paper, and fresh weight (*FW*) measured in laboratory, and then dried at 65 °C to constant weight. Dry weight (*DW*) was recorded. Water content was expressed as the percentage of moisture in FW. Biomass per unit area was calculated as:

*B* = *DW*/*A* (2)

where *B* is the biomass per unit area (g/m^2^), *DW* is the dry weight (g), and *A* is the sampling area (m^2^).

#### 2.2.4. Data Processing

Data analyses were primarily performed using R (v4.3.2) within RStudio (2023.12.0+369) with packages corrplot, ggplot2, linkET, ggcor, ggcorplot, and vegan. The correlation analyses were conducted to evaluate relationships between environmental parameters and both the coverage and maximum biomass of filamentous algae across sampling sites. In addition, a two-way analysis of variance (two-way ANOVA) was performed using Python (v3.11) to assess the effects of sampling time and site on filamentous algal coverage and biomass.

## 3. Results

### 3.1. Filamentous Algae Species Composition

#### 3.1.1. Morphological Identification

**Figure 2:**
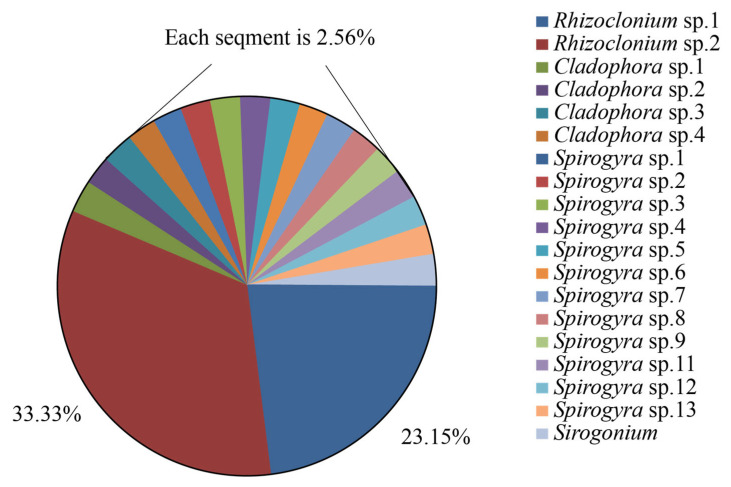
The relative frequency of filamentous algal taxa in *Eriocheir sinensis* aquaculture ponds on the Chongming Island.

During the sampling period, four genera of filamentous algae were identified: *Cladophora*, *Rhizoclonium*, *Spirogyra*, and *Sirogonium*; these genera represent two phyla, with *Cladophora* and *Rhizoclonium* belonging to Chlorophyta and *Spirogyra* and *Sirogonium* belonging to Charophyta. Based on the ITS sequences results, *Rhizoclonium* was the most frequently detected genus and was found in samples from all surveyed farms (Figure 2). It was consistently present throughout the study. *Spirogyra* and *Cladophora* were the next most frequently recorded genera, and *Spirogyra* was recorded in the Huikang, Yuxi, Baodao, and Yuhaitang farms.

**Figure 3:**
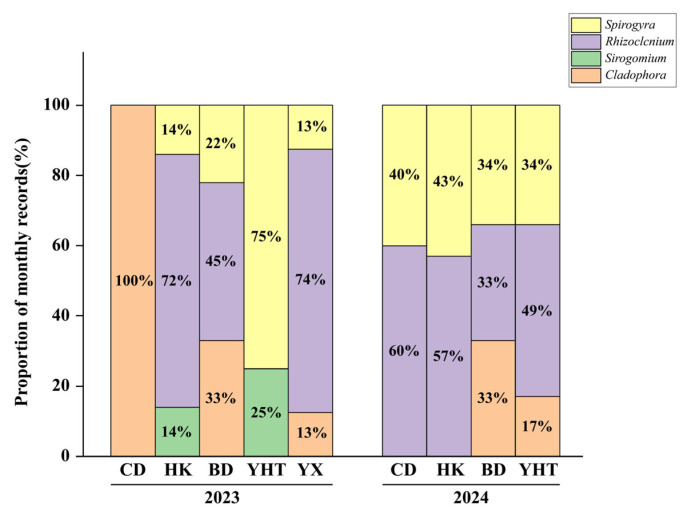
The proportions of monthly genus records of filamentous algae at each *Eriocheir sinensis* aquaculture farm in Chongming.

The Chongming *Eriocheir sinensis* aquaculture farms were divided into eastern regions (HK, CD, and YX) and western regions (BD and YHT) based on geographical location. In 2023, samples collected from Chongdong were identified as *Cladophora*. Based on the monthly survey records, *Rhizoclonium* was recorded most frequently in Huikang (72% of the monthly records). In Baodao, *Cladophora* accounted for 33% of the monthly records, whereas *Rhizoclonium* accounted for 45%. In Yuxi, *Cladophora* accounted for 13% of the records, while *Rhizoclonium* accounted for 74% (Figure 3).

In 2024, *Rhizoclonium* accounted for 60% of the monthly records in Chongdong, and *Spirogyra* accounted for 40%. In HuiKang, *Rhizoclonium* accounted for 57% of the monthly records. In Bao Dao, *Spirogyra*, *Rhizoclonium*, and *Cladophora* had similar occurrence frequencies (34%, 33%, and 33%, respectively). In Yuhai Tang, *Rhizoclonium* accounted for 49% of the monthly records. Across all sites, *Rhizoclonium* was the most frequently recorded genus.

#### 3.1.2. ITS Molecular Sequencing

**Figure 4:**
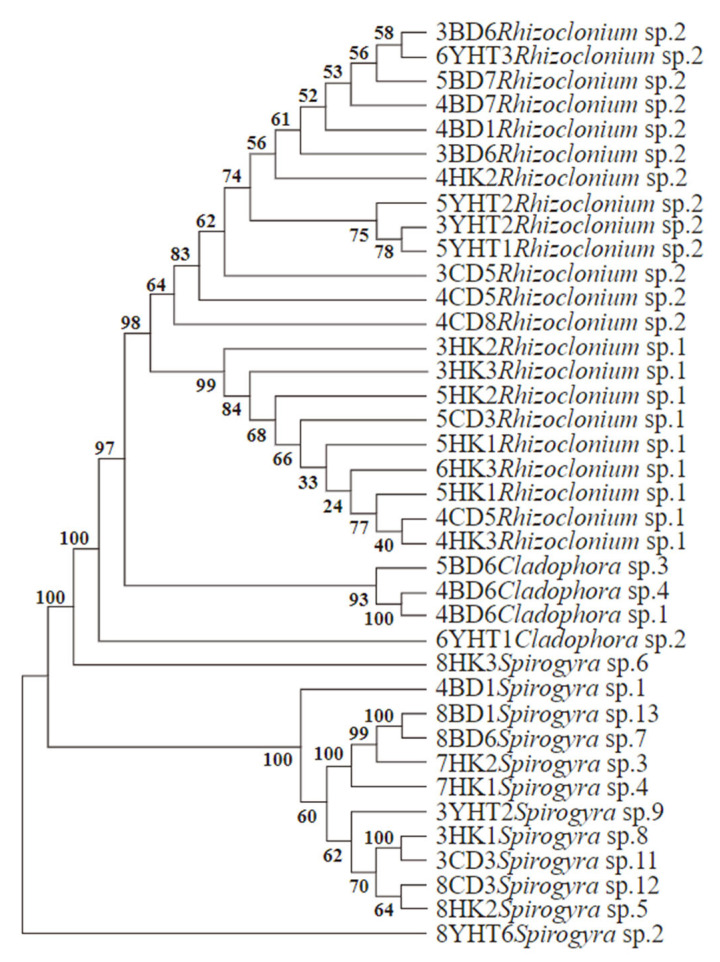
The phylogenetic tree for the ITS rDNA of the new species belong to filamentous algae. The values above branches indicate bootstrap values.

ITS sequences were obtained for *Rhizoclonium*, *Cladophora*, and *Spirogyra*; *Sirogonium* was identified morphologically and was therefore not included in the ITS-based phylogenetic analysis (Figure 4). The molecular identification results were consistent with morphological observations. The ITS rDNA sequences of the genus *Rhizoclonium* obtained in this study have been deposited in the NCBI database. A phylogenetic tree constructed from these sequences showed that *Rhizoclonium* formed multiple branches with genetic distances of 55.00~57.00%. *Cladophora* clustered independently in the middle of the tree, closely related with but distinct from *Rhizoclonium*, with genetic distances of 60.00~62.00%. *Spirogyra* formed a larger, more distant branch, with genetic distances of 71.00~77.00%. The relatively shorter branch lengths of *Rhizoclonium* and *Cladophora* indicated close genetic relationships and high support values, whereas *Spirogyra* exhibited longer branches and greater evolutionary divergence.

### 3.2. Biomass and Coverage of Filamentous Algae

The dry weight of filamentous algae samples ranged from 0.69 to 10.73 g, corresponding to a dry weight per unit area of 2.76~42.92 g/m^2^. The biomass values varied markedly across time and space, especially in May. The maximum biomass of filamentous algae was recorded in a sample collected from the Baodao Farm in May 2024, reaching a peak value of 42.92 g/m^2^.

**Figure 5:**
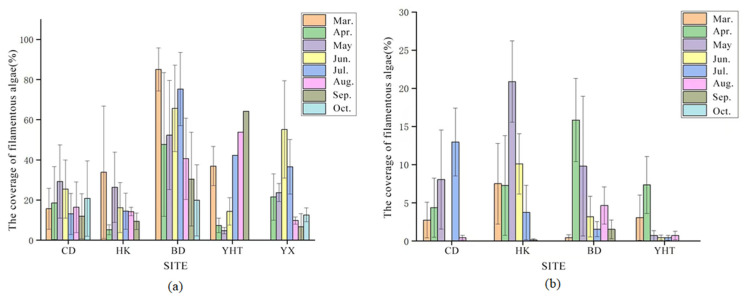
Filamentous algal coverage at *Eriocheir sinensis* farms in Chongming in (**a**) 2023 and (**b**) 2024.

Two-way ANOVA indicated significant effects of month and region on filamentous algal coverage and biomass, as well as a significant month × region interaction. In 2023, algal coverage exhibited substantial spatial and temporal variability (Figure 5a). No uniform seasonal pattern was observed across all farms. The Baodao Farm maintained high coverage from spring onward, while others, such as Yuhaitang, showed a decline during the same period. A summer peak was evident at some farms, confirming strong spatial heterogeneity, with western farms (Baodao) experiencing higher filamentous algal pressure than the eastern ones (Yuhaitang).

In 2024, algal coverage between March and May was significantly lower than in the previous year (Figure 5b). The coverage at the Chongdong farm, which fluctuated in 2023, dropped below 25% in 2024 and remained stable. Overall, the western farms (BD and YHT) of Chongming exhibited greater filamentous algal coverage than the eastern farms (HK, CD, and YX) in 2023.

### 3.3. The Relationship Between Filamentous Algal Biomass and Environmental Factors

#### 3.3.1. The Dynamics of the Water Environment Factor

The ranges of water environment parameters across different farms during the study period are presented in Table 2. Clear spatiotemporal patterns were observed. Year-to-year comparisons showed notable declines in mean concentrations of key nutrients (TN and NH_3_-N) from 2023 to 2024. Conversely, the permanganate index (COD_Mn_) increased at all sites in 2024, suggesting higher levels of oxidizable organic matter.

#### 3.3.2. The Relationship Between Algal Coverage and Environmental Variables

**Figure 6:**
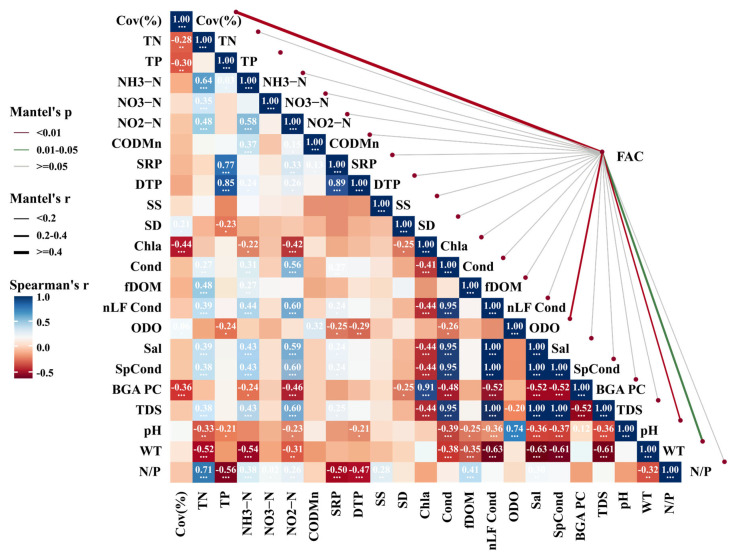
The relationships between filamentous algal coverage and environmental factors. (TP: total phosphorus; DTP: dissolved total phosphorus; SRP: soluble reactive phosphorus; TN: total nitrogen; NH_3_−N: ammonia nitrogen; NO_2_^−^−N: nitrite nitrogen; NO_3_^−^−N: nitrate nitrogen; N/P: nitrogen-to-phosphorus ratio; COD_Mn_: permanganate index; fDOM: fluorescent dissolved organic matter; Chl−a: chlorophyll-a; BGA pc: phycocyanin, RFU; ODO: dissolved oxygen; Cond: electrical conductivity; SpCond: specific conductivity; nLF Cond: normalized low-frequency conductivity; TDS: total dissolved solids; Sal: salinity; SS: suspended solids; SD: Secchi depth; WT: water temperature). (* *p* < 0.05, ** *p* < 0.01, *** *p* < 0.001; unmarked indicates *p* > 0.05 (not significant)).

**Figure 7:**
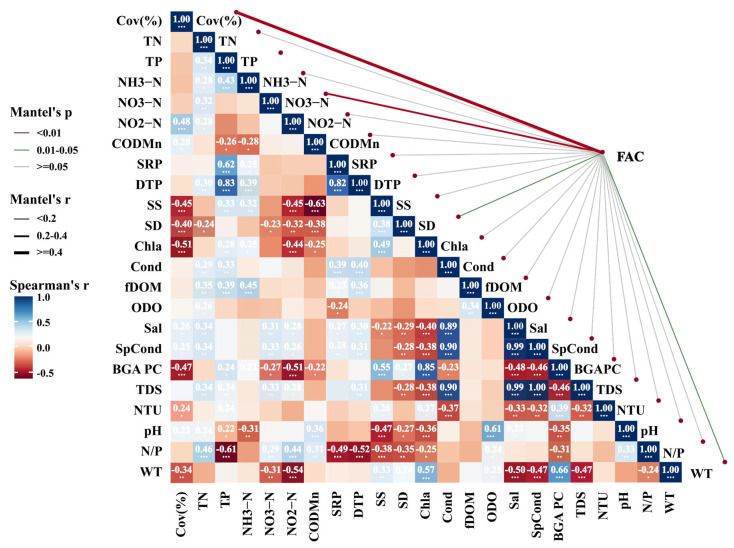
The relationships between filamentous algal coverage and prior-month environmental factors. (NTU: Nephelometric Turbidity Unit; other abbreviations are defined in Figure 6). (* *p* < 0.05, ** *p* < 0.01, *** *p* < 0.001); unmarked indicates *p* > 0.05 (not significant).

Coverage exhibited significant positive correlations with dissolved oxygen, pH, water temperature, transparency, and fluorescent dissolved organic matter (fDOM) (Figure 6), and a significant negative correlation with nitrate nitrogen concentrations from the preceding month (Figure 7). Among these variables, pH showed a relatively strong association. However, as pH both influences and is influenced by algal photosynthetic activity, these relationships likely reflect complex, interactive feedback within the aquatic system.

#### 3.3.3. The Relationship Between Algal Biomass and Environmental Factors

**Figure 8:**
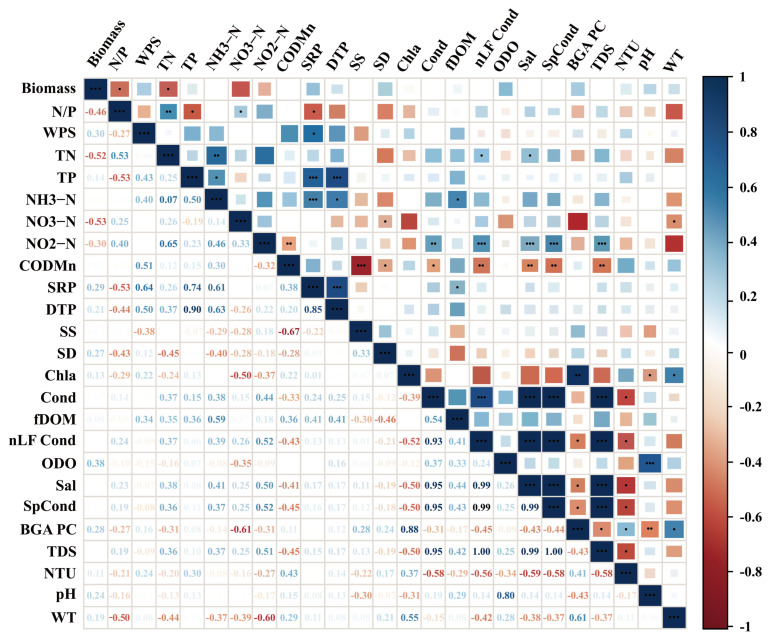
The relationship between filamentous algal biomass and environmental parameters in Chongming *Eriocheir sinensis* aquaculture ponds. (Abbreviations are defined in Figure 7) (* *p* < 0.05, ** *p* < 0.01, and *** *p* < 0.001); unmarked indicates *p* > 0.05 (not significant).

The Spearman correlation analyses (Figure 8) revealed that the total biomass of filamentous algae was negatively correlated with the nitrogen-to-phosphorus ratio (r = −0.46, *p* < 0.05) and total nitrogen (r = −0.52, *p* < 0.01); biomass also showed negative trends with ammonia nitrogen, nitrate nitrogen, and nitrite nitrogen, but these relationships were weak and not statistically significant (*p* > 0.05). Conversely, biomass showed weak positive associations with total phosphorus, soluble reactive phosphorus, dissolved total phosphorus, pH, and water temperature (all *p* > 0.05). The strongest correlation was found between algal biomass and nitrate nitrogen (r = −0.53, *p* < 0.01).

**Figure 9:**
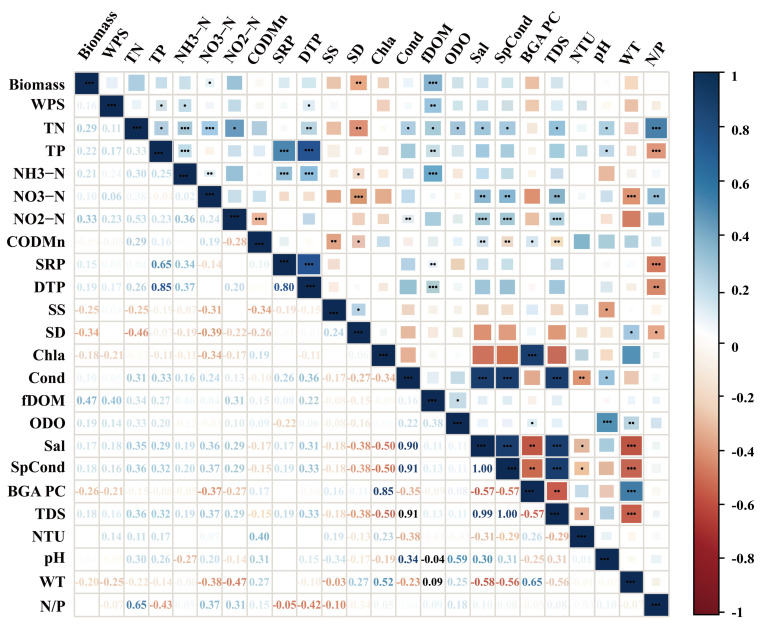
The relationship between filamentous algal biomass and prior-month environmental drivers in Chongming *Eriocheir sinensis* aquaculture ponds. (Abbreviations are defined in Figure 6) (Significant correlations are indicated by asterisks (* *p* < 0.05, ** *p* < 0.01, *** *p* < 0.001; unmarked indicates *p* > 0.05 (not significant))).

When analyzing lag effects (Figure 9), biomass showed positive correlations with total nitrogen, total phosphorus, ammonia nitrogen, nitrate nitrogen, nitrite nitrogen, soluble reactive phosphorus, and dissolved total phosphorus, with nitrate nitrogen again showing the strongest association. In contrast, suspended solids, transparency, chlorophyll a, and water temperature were negatively correlated with algal biomass.

**Figure 10:**
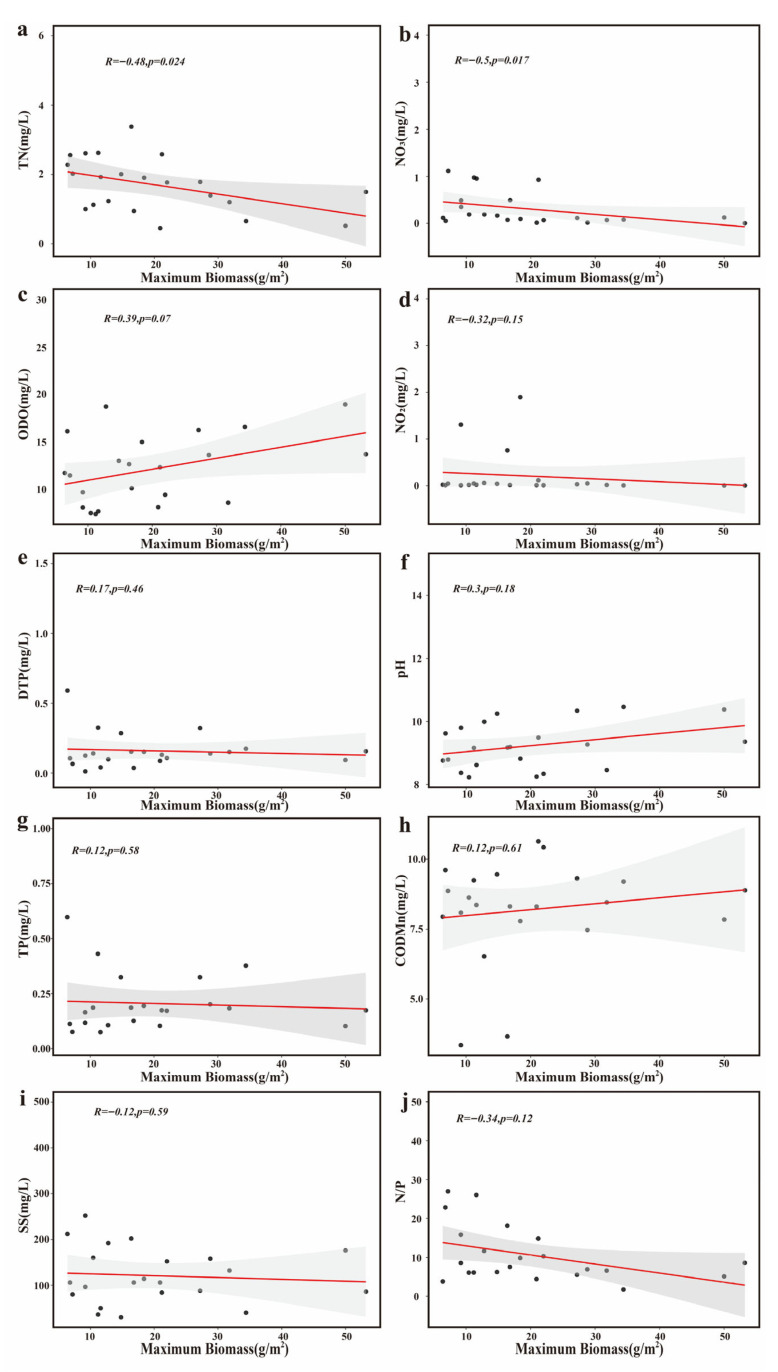
The correlation analysis between filamentous algal biomass and environmental factors in Chongming *Eriocheir sinensis* aquaculture ponds. (**a**) TN: total nitrogen; (**b**) NO_3_: nitrate nitrogen; (**c**) ODO: optical dissolved oxygen; (**d**) NO_2_: nitrite nitrogen; (**e**) DTP: dissolved total phosphorus; (**f**) pH: hydrogen ion activity; (**g**) TP: total phosphorus; (**h**) COD_Mn_: permanganate index; (**i**) SS: suspended solids; and (**j**) N/P: nitrogen-to-phosphorus ratio. (Each panel reports the correlation coefficient (*R*) and the corresponding *p* value. *p* < 0.05 indicates statistical significance).

Linear regression analyses (Figure 10) further demonstrated a significant negative correlation between the maximum filamentous algal biomass and both total nitrogen (R = −0.48, *p* = 0.024) and nitrate nitrogen (R = −0.50, *p* = 0.017), with nitrate nitrogen showing one of the strongest associations. Suspended solids showed a weak negative association, while total phosphorus was not significantly correlated. Based on these results and the relatively high total phosphorus concentrations recorded in these ponds, it is unlikely that phosphorus availability was the primary factor limiting filamentous algal growth. Dissolved oxygen showed a weak positive correlation with biomass, which is more plausibly attributable to increased daytime oxygen release from dense algal mats [24].

**Figure 11:**
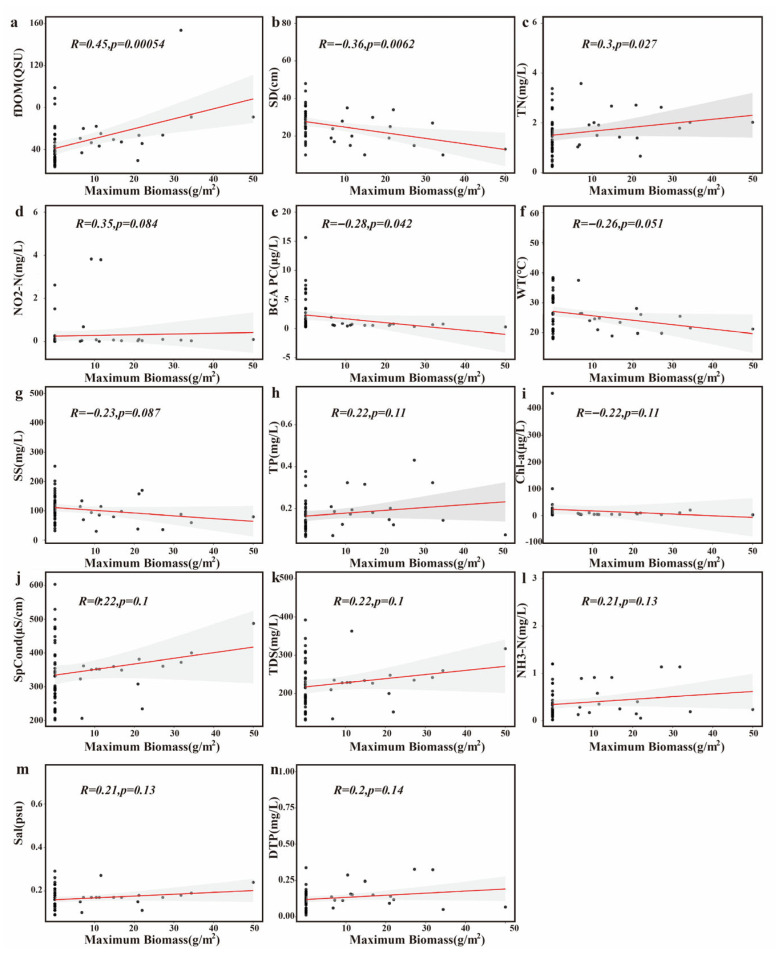
The correlation analysis between the maximum biomass of filamentous algae and the prior-month environmental factors. (**a**) fDOM: fluorescent dissolved organic matter; (**b**) SD: Secchi depth; (**c**) TN: total nitrogen; (**d**) NO_2_-N: nitrite nitrogen; (**e**) BGA PC: blue-green algae phycocyanin; (**f**) WT: water temperature; (**g**) SS: suspended solids; (**h**) TP: total phosphorus; (**i**) Chla: chlorophyll a; (**j**) SpCond: specific conductivity; (**k**) TDS: total dissolved solids; (**l**) NH_3_-N: ammonia nitrogen; (**m**) Sal: salinity; (**n**) DTP: dissolved total phosphorus. (Each panel reports the correlation coefficient (*R*) and the corresponding *p* value. *p* < 0.05 indicates statistical significance).

Lagged analyses (Figure 11) showed that fluorescent dissolved organic matter (fDOM) was positively associated with algal biomass (R = 0.45, *p* = 0.00054). Biomass was also positively associated with total nitrogen (R = 0.30, *p* = 0.027) and negatively associated with water transparency (R = −0.36, *p* = 0.0062). In these ponds, fDOM and ammonium nitrogen derive primarily from uneaten feed and metabolic waste of crabs and other animals, with additional inputs from extracellular organic matter released by algae and macrophytes. Water temperature was negatively related to biomass with marginal significance (R = −0.26, *p* = 0.051), whereas suspended solids, chlorophyll a, and phosphorus variables showed weak associations (all *p* > 0.05).

## 4. Discussion

Previous studies reported that most filamentous algal blooms in freshwater systems are dominated by *Spirogyra*, *Zygnema*, and *Cladophora* [25,26,27]. The filamentous algae recorded in this study were recorded primarily as *Rhizoclonium*, *Spirogyra*, and *Cladophora*, whereas *Sirogonium* was recorded infrequently. *Rhizoclonium* accounted for about half of all records at the genus level, followed by *Spirogyra* and *Cladophora*. *Cladophora* and *Spirogyra* are widely reported as common filamentous green algae in eutrophic rivers and lakes, whereas *Rhizoclonium* has received comparatively less attention [28]. In contrast, our survey records indicate that *Rhizoclonium* was the most frequently recorded genus in *E. sinensis* aquaculture ponds, and this pattern was consistent across farms and years. While the filamentous algae genera in *E. sinensis* ponds overlap with those in other eutrophic freshwater habitats, their relative frequencies are shaped by the shallow water depth, abundant macrophytes, and high organic loading characteristic of the *E. sinensis* aquaculture.

Due to high morphological variability, species identification within filamentous algae is challenging. Marks and Cummings [29] first used ITS to identify freshwater *Cladophora* from different habitats. Our ITS rDNA phylogeny recovered three well-supported clades corresponding to *Rhizoclonium*, *Cladophora*, and *Spirogyra*, with short branch lengths and high bootstrap support within the *Rhizoclonium* and *Cladophora* clades and longer internal branches in *Spirogyra*. The phylogenetic analysis revealed several closely related local lineages within each genus. We found that multiple haplotypes or closely related taxa coexist in the aquaculture ponds, reflecting a high level of intraspecific or cryptic genetic diversity among filamentous algal populations. The current taxonomic information on filamentous algae remains limited. Community composition is diverse, interspecific morphological differences are often subtle, and diagnostic characters vary with ontogenetic stage and environmental conditions [30]. Consequently, identification based on morphology remains challenging, and substantial additional work is required to achieve reliable species-level determinations of filamentous algae.

Time series records of filamentous algal coverage and biomass revealed complex spatiotemporal patterns across *Eriocheir sinensis* ponds on Chongming Island, similar to patterns observed in natural and semi-natural freshwater systems [31,32], consistent with the high spatial and temporal variability commonly reported for periphytic algal communities. In 2023, the timing and magnitude of coverage peaks differed markedly among farms, and, in 2024, the overall coverage was substantially lower at all farms and no consistent peak period emerged, indicating pronounced interannual variability in bloom intensity [33].

Filamentous algal biomass dynamics showed a similarly heterogeneous pattern. Across ponds, filamentous algal biomass generally increased in spring, often reaching a spring maximum, followed by variable trajectories in summer and autumn. Comparable seasonal fluctuations in periphyton community biomass and nutrient status have been documented in reservoirs of both natural macrophytes and artificial substrates [34], suggesting that seasonal variability is characteristic of attached algal communities. In some ponds, the biomass declined sharply after the spring peak, likely reflecting nutrient limitation or shading caused by dense algal growth. In addition to environmental factors, variation in pond management practices may have influenced the differences in biomass trajectories. In the YHT and HK *E. sinensis* ponds, filamentous algal biomass peaked in May 2024, and then declined sharply, coinciding with the application of algicides for filamentous algal control. In other ponds, the biomass remained moderate or showed secondary increases later in the growing season, a pattern also reported in regard to attached filamentous algal communities in extended records from temperate lakes [33]. Such asynchronous seasonal responses suggest that local environmental heterogeneity, including light, substrate stability, and nutrient supply, can strongly modulate biomass trajectories across sites [35].

The lagged correlation analysis further suggested that filamentous algal biomass integrates environmental conditions over time, rather than responding solely to instantaneous water conditions. Villeneuve [36] showed that benthic algae act as integrators of light, as well as flow and nutrients, over several weeks, which supports the interpretation that filamentous algal biomass reflects accumulated environmental effects. Biomass showed relatively strong associations with indicators of nutrient and organic enrichment, such as fluorescent dissolved organic matter (fDOM), nitrite, and ammonia nitrogen, when these variables were considered with a time lag of one to several weeks; comparable delayed responses of benthic communities to nutrient and organic inputs have been documented in lakes [37]. These time lags suggest that opportunistic filamentous algae can rapidly exploit favorable conditions, whereas the formation of thick mats and feedback processes tends to occur more gradually over several weeks [38].

Light, temperature, and nutrients have strong direct effects on the growth of attached filamentous algae [39]. The correlation and regression analyses indicated that several physicochemical variables were closely associated with the occurrence of filamentous algae in the *Eriocheir sinensis* ponds, reflecting the combined influence of nutrient enrichment and light availability, as modulated by water transparency, on attached filamentous algal development [40]. Among the measured parameters, pH showed a positive correlation with filamentous algal coverage, consistent with reports from freshwater systems in which elevated pH, nutrient enrichment, and stable substrates favor filamentous mat formation [41]. Coverage showed negative trends with turbidity and suspended solids, whereas maximum biomass, defined as the highest quadrat value within a pond on each sampling date to represent the local peak standing crop, was associated with indicators of nutrient and organic enrichment and with submerged macrophyte coverage, echoing observations that nutrient and organic inputs, fine sediment, and aquatic vegetation interact to enhance filamentous algal mats [42].

The positive association between pH and filamentous algal coverage likely reflects a combination of environmental conditions and algal feedback on water chemistry [43]. Dense algal mats can elevate daytime pH by consuming dissolved CO_2_ through photosynthesis [44], while nocturnal respiration can drive pH back toward lower values [45]. Under the slightly alkaline conditions typical of these ponds, bicarbonate becomes an important inorganic carbon source that many filamentous algae can utilize [46]. We found that the positive link between pH and filamentous algal abundance arises from both favorable alkaline conditions and photosynthetically-driven feedbacks that further elevate pH in dense mats.

Temperature also influenced algal dynamics indirectly by modulating metabolic rates and nutrient transformations [47]. Although higher temperatures in aquaculture ponds are typically accompanied by increased feeding, and thus greater potential nitrogen inputs, the observed negative correlations between temperature, TN, and ammonium indicate lower measured nitrogen concentrations under warmer conditions. Faster microbial mineralization and biological uptake may increase nitrogen turnover and partially offset inputs, and enhanced macrophyte growth under warmer conditions may further draw down dissolved inorganic nitrogen, thereby altering nitrogen availability to filamentous algae [48].

Light availability, as reflected by turbidity and chlorophyll a, played a key role in determining where filamentous mats could expand [49]. Previous studies have noted that some species of *Cladophora* generally prefer high light and shallow water habitats [50]. Coverage showed a negative association with turbidity (*p* > 0.05), suggesting that elevated suspended particles and phytoplankton may reduce light penetration and potentially limit filamentous algal development [51]. Chlorophyll a concentrations, an indicator of phytoplankton biomass, were negatively associated with filamentous algal coverage (R = −0.44, *p* < 0.001), which may reflect resource competition, particularly for light, between phytoplankton and filamentous algae. Under highly turbid conditions, shading of the benthos likely suppresses the establishment or persistence of thick filamentous mats, even when nutrients are abundant [52].

Filamentous algal biomass showed consistent associations with nutrient concentrations, with the nitrogen-to-phosphorus (N/P) ratio serving as a key stoichiometric indicator [53]. Biomass was negatively correlated with N/P ratio (R = −0.34, *p* = 0.12). Filamentous mats developed more readily under conditions of abundant phosphorus and moderate nitrogen, whereas disproportionately high N/P ratios coincided with lower biomass levels [54]. The regression and lagged analyses further underscored the role of nutrient balance: the N/P ratio was a stronger predictor of biomass than either nutrient alone, and prior nutrient levels were positively associated with subsequent algal accumulation [39,55]. These patterns suggest a gradual biomass response to sustained nutrient enrichment, rather than an immediate reaction to short-term fluctuations.

Indicators of organic enrichment exhibited strong and often delayed correlations with filamentous algal biomass [56,57]. The permanganate index (COD_Mn_) showed a weak positive association with biomass (R = 0.12, *p* = 0.61), whereas fDOM was significantly and positively associated with biomass (R = 0.45, *p* = 0.00054), particularly when considered with a time lag of one or more sampling intervals [58]. These variables integrate dissolved and particulate organic matter derived from uneaten feed, feces, and excreta of crabs and other animals, as well as extracellular organic matter released by algae and macrophytes, together with resuspension of organic matter from sediments [59]. Continued organic enrichment and progressive mineralization can increase nutrient availability over time, creating conditions conducive to the gradual accumulation of filamentous mats rather than an instantaneous bloom [60]. At the same time, high organic loads can drive pronounced diel fluctuations in dissolved oxygen through enhanced community respiration, which may give feedback on algal physiology and community structure [45,61].

These results provide implications for pond management. Filamentous algal biomass was associated with cumulative nutrient and organic inputs and showed lagged responses, suggesting that preventive management targeting feed loss and organic matter accumulation, together with nitrogen management, may help reduce the risk of filamentous algal proliferation in *Eriocheir sinensis* ponds. However, we did not test specific control interventions, and the relative effectiveness of preventive measures versus chemical suppression should be evaluated in dedicated field experiments with appropriate controls. Maintaining an appropriate nitrogen-to-phosphorus balance, improving feeding practices, and restricting excessive organic inputs from routine operations may help sustain filamentous algal development. In addition, regulating light availability by managing turbidity, controlling filamentous algal biomass, and keeping submerged macrophyte coverage within suitable bounds may further reduce conditions that favor filamentous algal mats.

## 5. Conclusions

Filamentous algal biomass in *Eriocheir sinensis* aquaculture ponds exhibited pronounced seasonal variability, reflecting strong temporal dynamics under nutrient-rich conditions. Biomass was regulated primarily through nutrient balance, organic loading, and light conditions acting jointly over time. In particular, nitrogen stoichiometry and indicators of organic enrichment played a central role in shaping biomass trajectories, and the clear time lag in these responses indicated that algal blooms integrated cumulative environmental pressures rather than instantaneous water quality conditions. From a management perspective, the results indicated that an effective control of filamentous algae in *Eriocheir sinensis* aquaculture ponds should prioritize preventive, ecologically-based strategies. Reducing excessive nutrient and organic inputs, maintaining balanced nutrient ratios, and regulating light availability through integrated pond management are likely to be more effective and sustainable than reactive chemical suppression.

## Figures and Tables

**Table 1:** The basic information on *Eriocheir sinensis* aquaculture ponds in Chongming.

Number of Pond	Aquaculture Farms	Self-Designation	Sampling Year	Area of Pond/667 m^2^	Stocking Density/(ind/667 m^2^)	Stocking Model	Planted Macrophytes	Management
1	Chongdong	CD3	2023/ 2024	35	1000	Mixed male and female	*Elodea canadensis*	Regular manual removal of filamentous algae/non-chemical control
2		CD5		25	1000	Male only	*Elodea canadensis*	
3		CD8		25	1000	Female only	*Elodea canadensis*	
4	Huikang	HK1	2023/ 2024	11.4	2500	Mixed male and female	*Elodea canadensis* + 1/3 *Ceratophyllum demersum*	Regular manual removal of filamentous algae/application of potassium fulvate and sodium humate at 0.5 kg per 667 m^2^, applied once every 8–10 days
5		HK2/HK1		7.9	1200	Mixed male and female	*Elodea canadensis* + 1/2 *Ceratophyllum demersum*	
6		HK3/HK2		7.8	1200	Male only	*Elodea canadensis* + 1/2 *Ceratophyllum demersum*	
7		HK3		7.9	1200	Female only	*Elodea canadensis* + 1/2 *Ceratophyllum demersum*	
8	Baodao	BD1	2023/ 2024	13.4	1000	Mixed male and female	*Elodea canadensis*	Regular manual removal of filamentous algae/non-chemical control
9		BD2		25.6	1000	Mixed male and female	*Elodea canadensis*	
10		BD6		13	1000	Female only	*Elodea canadensis*	
11		BD7		15	1000	Male only	*Elodea canadensis*	
12	Yuhaitang	YHT1	2023/ 2024	5.1	1250	No crab seedlings	Rice in the middle, *Elodea canadensis* around	Regular manual removal of filamentous algae/application of potassium fulvate and sodium humate at 0.5 kg per 667 m^2^, applied twice a month
13		YHT2		15.6	1250	With crab seedlings	Rice in the middle, *Elodea canadensis* around	
14		YHT3		28.3	800	With crab seedlings	Rice in the middle, *Elodea canadensis* around	
15		YHT1		16.06	1050	Female only	Pellet feed, cornmeal	
16		YHT2		18.4	1200	Female only	Pellet feed, cornmeal	
17		YHT6		12.25	1400	Mixed male and female	Sodium humate, humic acid sodium for algae control	
18	Yuxi	YX2	2023	8	1000	Mixed male and female	*Alternanthera philoxeroides* + *Elodea canadensis*	Regular manual removal of filamentous algae/non-chemical control
19		YX10		10	640	Female only	*Elodea canadensis* + *Ceratophyllum demersum*	

**Table 2:** The annual variations in water quality parameters of *Eriocheir sinensis* aquaculture ponds in Chongming.

Factor	Year	Chongdong (Mean ± SD)	Huikang (Mean ± SD)	Baodao (Mean ± SD)	Yuhaitang (Mean ± SD)	Yuxi (Mean ± SD)
TN (mg/L)	2023	2.53 ± 2.17	5.45 ± 5.20	2.08 ± 1.44	4.50 ± 5.00	2.38 ± 1.06
	2024	1.71 ± 1.09	2.22 ± 1.10	1.62 ± 0.71	1.56 ± 0.65	NA
TP (mg/L)	2023	0.31 ± 0.17	0.52 ± 0.41	0.20 ± 0.09	0.17 ± 0.08	0.28 ± 0.25
	2024	0.23 ± 0.16	0.32 ± 0.19	0.24 ± 0.16	0.14 ± 0.05	NA
NH_3_-N (mg/L)	2023	0.44 ± 0.33	2.28 ± 2.61	0.38 ± 0.52	1.78 ± 3.32	0.3 ± 0.27
	2024	0.38 ± 0.23	0.75 ± 0.71	0.44 ± 0.24	0.43 ± 0.39	NA
COD_Mn_	2023	4.34 ± 1.76	5.64 ± 1.94	4.87 ± 1.66	4.98 ± 1.43	4.87 ± 1.79
	2024	7.73 ± 1.91	8.76 ± 1.34	8.21 ± 1.43	7.90 ± 2.02	NA
DO (mg/L)	2023	6.99 ± 2.00	9.42 ± 2.66	11.84 ± 3.39	11.13 ± 2.80	7.84 ± 2.19
	2024	7.13 ± 2.30	11.10 ± 3.26	11.82 ± 3.79	12.81 ± 3.97	NA

Abbreviations: TN—total nitrogen; TP—total phosphorus; NH_3_-N—ammonia nitrogen; COD_Mn_—permanganate index; DO—dissolved oxygen; NA—not available.

## Data Availability

The data presented in this study are available upon request from the corresponding author.

## Abbreviations

The following abbreviations were used in this manuscript:

CD	Chongdong Aquaculture Farm
HK	Huikang Aquaculture Farm
YHT	Yuhaitang Aquaculture Farm
BD	Baodao Farm
YX	Yuxi Aquaculture Farm
